# Could Fahr’s Syndrome Have More Than One Simultaneous Etiology?

**DOI:** 10.7759/cureus.20342

**Published:** 2021-12-11

**Authors:** Gabriele Palu, Samia T Moraes, Gabriela Romaniello, Luis O Zatorre, Luiza K Seixas, Rafael Miyazima, Gustavo Lenci Marques

**Affiliations:** 1 Internal Medicine Department, Federal University of Parana, Curitiba, BRA; 2 Medicine Department, Pontifical Catholic University of Paraná, Curitiba, BRA

**Keywords:** secondary hypocalcemia, cerebral calcifications, new-onset seizure, hypoparathyroidism, fahr’s disease or fahr’s syndrome

## Abstract

Fahr's syndrome is a rare, genetically dominant, inherited, neurological disorder characterized by abnormal deposits of calcium in the basal ganglia and the cerebral cortex. Symptoms include motor dysfunction, dementia, headache, spastic paralysis, abnormal ocular findings and seizures. Hypoparathyroidism is the most common endocrine disorder related to this syndrome, however, there are other metabolic, infectious and genetic causes. This is a case report of a Fahr’s syndrome patient presenting a three-month history of self-limited partial epileptic seizures. His cranial CT had bilateral symmetrical calcifications of the basal ganglia, subcortical tissue and dentate nucleus whereas his laboratory findings were compatible with hypoparathyroidism.

## Introduction

Basal ganglia calcifications could be “physiological” (such as in patients over 50 years) but also an incidental CT finding in 15-20% of asymptomatic patients [1]. However, in less than 1/1,000,000 of cases, it has clinical significance, being associated with an extremely rare disorder characterized by bilateral symmetrical calcifications of the basal ganglia and the cerebral cortex, denominated as Fahr's syndrome [2, 3].

The term “syndrome” should be used to reflect the clinical and radiological picture, when there is a secondary etiology responsible for the findings. On the other hand, idiopathic calcifications have traditionally been described as Fahr's disease, based on Theodor Fahr’s report “idiopathic calcification of cerebral vessels” [4]. Fahr's syndrome is characterized, histologically, by foci of symmetrical, non-atheromatic compounds that are located within the globus pallidus, striatum, dentate nucleus, basal ganglia as well as within white and gray matter of the brain and cerebellum. The deposits consist mainly of calcium, but it is also associated with the deposition of zinc, iron, aluminum, magnesium, silicon, copper and phosphorus [5].

The clinical presentation of Fahr's syndrome may involve neuropsychiatric disorders, cognitive impairment, fatigue, impaired gait, speech dysfunction, dysphagia, involuntary movements, cramps, dementia, extrapyramidal features, generalized or partial seizures, and rarely, pyramidal syndrome and intracranial hypertension [6,7]. Fahr's syndrome can be due to metabolic, infectious and genetic causes. The metabolic causes include hypoparathyroidism, pseudohypoparathyroidism, hypothyroidism, toxins such as lead and hypervitaminosis D. The infectious causes include toxoplasmosis, rubella, cytomegalovirus, neurocysticercosis, HIV and tuberculosis. It could also be due to rarer genetic disorders such as neurofibromatosis, tuberous sclerosis, Cockayne syndrome, Wilson’s disease [3]. An important differential diagnosis is idiopathic calcifications, denominated as Fahr's disease. This condition is most commonly found to be inherited in an autosomal dominant pattern with incomplete and age-related penetrance, but it may also be transmitted as an autosomal recessive trait or occur sporadically [8].

Hypoparathyroidism is the most common endocrine disorder related to Fahr's syndrome [1]. It is characterized by corrected serum calcium or ionized calcium concentration below the normal range, accompanied by undetectable or inappropriately low levels of parathyroid hormone (PTH). The combination of a low PTH level and a low serum calcium concentration is abnormal. Under physiological conditions, hypocalcemia will lead to an increase in PTH. The lack of PTH also leads to hyperphosphatemia because the phosphaturic actions of PTH are lost. It is important to ascertain that the serum magnesium level is normal because both hypermagnesemia and severe hypomagnesemia can lead to functional hypoparathyroidism [9]. The most common cause of hypoparathyroidism is inadvertent removal of, or injury to, the parathyroid glands during neck surgery, followed by genetic, idiopathic and autoimmune etiologies. Conventional treatment includes activated vitamin D and/or calcium supplements [9]. This case report is about a patient with Fahr's syndrome and hypoparathyroidism with no history of neck surgery or trauma, and initially unresponsive to magnesium replacement, presenting as a diagnostic challenge.

## Case presentation

A 70-year-old male patient was referred to a tertiary healthcare center (HC-UFPR), in August 2021, for evaluation of a three-month history of self-limited partial epileptic seizures, witnessed by his son and daughter-in-law. Previously, the patient was hospitalized at a primary hospital, in the southern Brazilian countryside (Mandirituba/PR) after a seizure, described as hypertonic, associated with loss of consciousness and a postictal state, without any involuntary spontaneous micturition. An increase of the QT interval of the EKG was also reported in the referral of that hospital.

The patient had a medical history of Barret’s esophagus and a surgery for cataract correction 20 years prior to the admission. He was a former smoker (20 pack-years) and also a former alcoholic. His family medical history included a son with epilepsy and a sister with an unknown disease that led to hospitalization with respiratory assistance. Physical examination included skin xerosis, digital clubbing, dry, brittle nails and a non-palpable thyroid. Oral examination revealed complete edentulism. The neurological examination presented a discretely photoreactive left pupil. The Chvostek and Trousseau signs were also present. The complete blood count revealed a microcytic and hypochromic anemia. The biochemical test results were: a total calcium of 4.5 mg/dL (Reference range: 8.5 - 10 mg/dL), albumin 3.6 mg/dL (Reference range: 2 - 4 mg/dL), ionized calcium 0.74 mmol/L (Reference range: 4.4 - 5.2 mmol/L), magnesium 1.5 mg/dL (Reference range: 1.7 - 2.2 mg/dL), phosphorus 6.7 mg/dL (Reference range: 3 - 4.5 mg/dL), PTH below 3.0 pg/mL (Reference range: 14 - 65 pg/mL) and the 24-hour urinary calcium 2.6 mg (Reference range: 100 - 300 mg).

**Figure 1:**
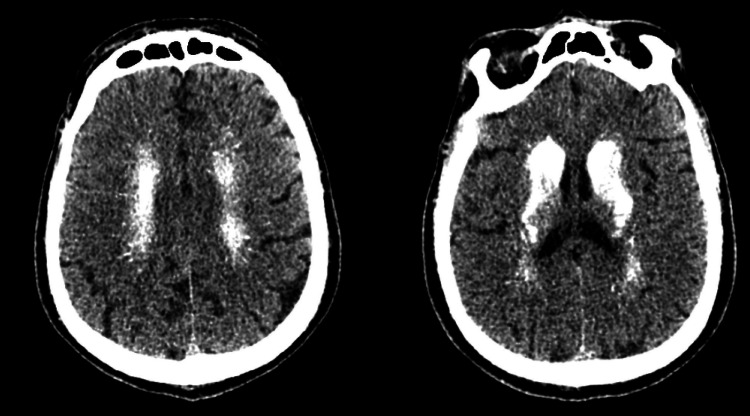
Cranial CT demonstrating important calcification in subcortical tissues.

**Figure 2:**
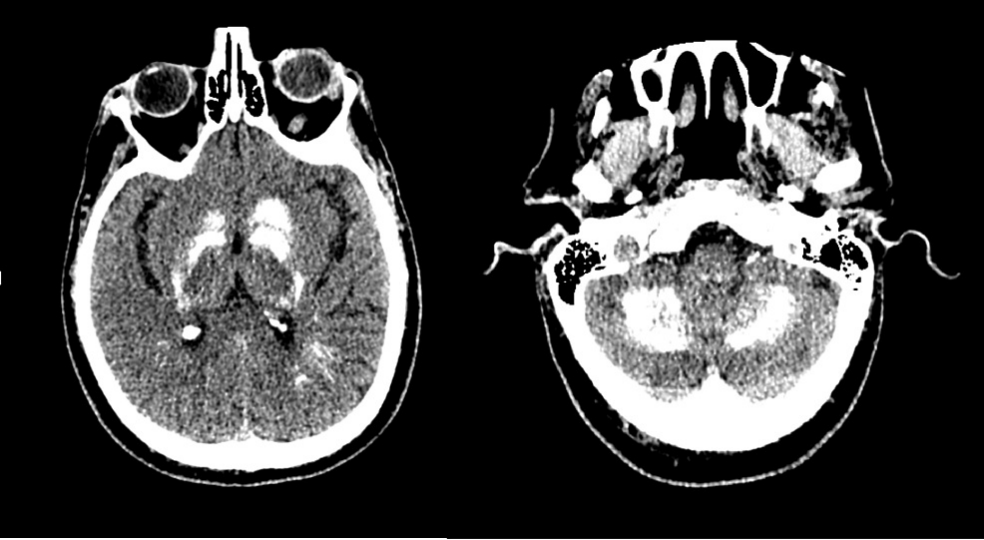
Cranial CT demonstrating important calcification in the basal ganglia and cerebellum.

The cranial CT presented bilateral symmetrical calcification of basal ganglia, subcortical tissue, and dentate nucleus (Figure 1 and Figure 2).

Electrolyte management was started with IV calcium and magnesium. Once the plasma calcium levels were corrected over 7.5 mg/dL, IV calcium was replaced by oral calcium carbonate, aiming at reducing phosphate levels, as well as oral vitamin D and calcitriol. The evaluation of causes and consequences of this hypoparathyroidism was to be investigated with an electroencephalogram, a thyroid/parathyroid ultrasound, a urinary system ultrasound, a transthoracic echocardiogram, X-rays of chest and knees, besides a thorax CT. Assessment of anti-calcium sensing receptor (CaSR) antibodies and genetic testing for Glial Cell Missing-2 (GCM2) would also be appropriate to do to rule out autoimmune disease, due to its high prevalence in hypoparathyroidism. However, the patient got a COVID-19 infection, most probably in the healthcare setting, worsening after three days of the diagnosis of the infection. He was mechanically ventilated and, although electrolyte levels were normal, he had bacterial infection complications, receiving care in the Intensive Care Unit.

## Discussion

The case describes an unusual presentation of Fahr’s syndrome, due to the underlying hypoparathyroidism associated with clonic-tonic seizures. The manifestation probably occurred not only because of the subcortical calcifications typical of Fahr’s syndrome - and found on the patient’s cranial CT (Figure 1 and Figure 2) - but mainly because of severe hypocalcemia secondary to the confirmed hypoparathyroidism [10].

The chief complaint of this patient was seizures. The investigation comprised of the biochemical evaluations and a cranial CT. The findings of serum calcium of 4.5 mg/dL, cranial CT basal ganglia and subcortical calcifications associated with an elevation of serum phosphorus made it imperative to test PTH serum levels, which were almost undetectable in the clinical case reported. The differences in Fahr’s disease and Fahr’s syndrome clinical presentations arise from the distinct etiologies of these entities, since Fahr’s disease is a primary idiopathic disorder and Fahr’s syndrome is secondary to a variety of metabolic alterations and diseases [4].

Among the causes of Fahr’s syndrome, the leading one is hypoparathyroidism which, in general, presents with intracranial calcifications that surpass the basal ganglia, compromising also cortical portions of the brain [1, 11] - a fact that was shown in this patient’s cranial images. The mechanism of cerebral calcifications in hypoparathyroidism is not completely understood. One of the proposed and most acceptable explanations is the association of cerebral calcifications and hyperphosphatemia, which leads to calcium vs phosphorus product elevation and, consequently, basal ganglia and cortical impairment by deposition [12, 13]. Another proposed cause is an increased expression of osteogenic molecules, such as osteonectin or osteopontin, especially in the caudate nucleus in the setting of hypoparathyroidism [14].

Inadvertent resection or iatrogenic damage of the parathyroid glands during neck procedures and surgeries are the major causes of hypoparathyroidism, accounting for 75% of the cases [15]. Autoimmune disorders of parathyroid glands, isolated or in the spectrum of polyglandular syndromes, are the second most common cause for hypoparathyroidism [16]. Variations of serum magnesium levels, both hyper and hypomagnesemia, may deregulate secretion of PTH, culminating in a reversible hypoparathyroidism [17]. Infiltrative diseases such as hemochromatosis and sarcoidosis, tumor metastasis and actinic lesion of the gland account for a small proportion of hypoparathyroidism causes [17, 18].

The chief endocrine disturbance of hypoparathyroidism is PTH deficiency, resulting in reduced production of active vitamin D, and, consequently, diminished intestinal calcium absorption and serum hypocalcemia. Treatment of the condition is, therefore, irrespectively of its etiology, centered on calcium and vitamin D reposition. When extremely low serum calcium levels are identified, intravenous calcium reposition is necessary because of the higher risk of cardiac arrhythmias and seizures [10, 18, 19]. The accurate diagnosis of the cause of hypoparathyroidism in this scenario is essential to prevent further episodes of acute hypocalcemia and chronic sequelae, especially, in the brains and kidneys, which may lead to increased morbidity [20]. In this case report, further investigation will occur in the outpatient setting, since its treatment has to be slowly adjusted and there's a limitation of resources to investigate in the inpatient setting.

## Conclusions

Fahr’s syndrome is a rare condition, requiring high suspicion to diagnose. It is uncertain how each possible etiology, including Fahr’s disease, plays a role in the pathophysiology of the condition, and what are the main therapeutic goals to be sought after its diagnosis. The presentation of a patient with new-onset seizures not responding to antiepileptic drugs seems to be a possible symptom to guide the investigation. This symptom is frequently investigated by a cranial CT to exclude many of the diseases causing new-onset seizures and therefore, it will frequently show calcifications. This finding of calcifications, in this case scenario of neurological disorders, will present as a diagnostic clue to further investigate Fahr’s syndrome, even though calcifications may be idiopathic. Metabolic, infectious and genetic causes must be in the clinician repertoire when faced with this radiologic finding.

The syndrome is also an underreported and understudied condition, which requires further research to show its best diagnostic and therapeutic approaches. When a neurological patient presents with brain calcifications, aiming to diagnose the condition and improve care seems to be the main goal, but it is also important not to iatrogenize idiopathic brain calcifications, due to its much larger prevalence than Fahr’s syndrome. Further investigation of hypoparathyroidism and all its possible causes seem to be a very reasonable choice in this patient, even though it is not totally certain the relationship between hypoparathyroidism and Fahr’s disease, and if this investigation improves the patient’s outcome.
